# Trifunctional antibody fusion proteins for targeted co-delivery of 4-1BBL with IL-15 or IL-21 to enhance antitumor immune responses

**DOI:** 10.3389/fimmu.2026.1903525

**Published:** 2026-10-07

**Authors:** Annika M. Möller, Felix Baumann, Max Pütter, Meng Xie, Ann-Kathrin Löffler, Dafne Müller

**Affiliations:** Institute of Cell Biology and Immunology, University of Stuttgart, Stuttgart, Germany

**Keywords:** 4-1BBL, antibody fusion proteins, cancer immunotherapy, IL-15, IL-21, trifunctional

## Abstract

Antibody fusion proteins incorporating immunomodulatory molecules, such as cytokines of the common γ-chain receptor family and costimulatory members of the TNF superfamily, hold great potential for cancer immunotherapy, with many candidates already advancing into clinical studies. However, the efficacy as single agent with one payload is limited and combinatorial approaches are required to fully exploit their immunomodulatory potential. Here, we describe a novel format of a trifunctional antibody fusion protein, composed of a tumor-directed Fab antibody fragment, specific for the fibroblast activation protein (FAP), fused to single-chain 4-1BBL in combination with either IL-15 connected to an IL-15Rα fragment or IL-21. Binding to target cells was confirmed, and activity was assessed on activated PBMCs either bound to target cells or in an untargeted setting. Combinatorial effects enhancing IFN-γ release were found to be targeting-dependent for both trifunctional antibody fusion proteins and superior to the activity observed with combined bifunctional antibody-fusion proteins carrying single payloads. In contrast, significant combined activity enhancing granzyme B expression was observed only for the trifunctional fusion protein containing sc4-1BBL/IL-21 and appeared to be targeting-independent. In 3D coculture settings of fibroblast spheroids and PBMCs, both trifunctional fusion proteins enhanced the cytotoxic effect of an EGFR-directed T cell engager (TCE) and cetuximab (CTX), whereby sc4-1BBL/IL-15 was more effective than sc4-1BBL/IL-21, and comparable effects were obtained by the respective bifunctional antibody-cytokines. The activity of antibody fusion proteins containing IL-15 was associated with strong activation of CD8+T cells, CD3+CD56+ T cells and NK cells, as well as enhanced proliferation, particularly in combination with TCE, expanding CD8+ T cells and NKT-like cells. Activation by fusion proteins containing IL-21 became mainly apparent in combination with TCE on CD8+ T cells and with CTX on NK cells, while proliferation was restricted to NK cells. Thus, the trifunctional antibody fusion proteins presenting sc4-1BBL/IL-15 and sc4-1BBL/IL-21 exhibit distinct patterns of combined activity and immune cell stimulation, both demonstrating combinatorial potential with TCE and CTX.

## 1 Introduction

The therapeutic potential of cytokines from the common γ-chain receptor family (e.g., IL-15 and IL-21) and agonists of costimulatory members of the TNFR superfamily (e.g., 4-1BBL) has been recognized for cancer immunotherapy (1, 2). However, clinical applications are challenged by short half-life, systemic toxicity, and limited efficacy when used as single agents. Development of tumor-directed antibody-fusion proteins address these limitations by extending half-life and enabling effective concentrations at lower doses through target-specific accumulation at the tumor site for cytokines, and targeting-dependent activity-on-demand for costimulatory ligands. Current efforts are focused on improving delivery strategies, modulating activity of single-payloads, and exploring combinatorial strategies to enhance antitumor immune responses (3–5).

Antibody fusion proteins incorporating IL-15 or IL-21 exert antitumor effects primarily by driving the expansion, survival, and enhancement of effector functions of CD8+ T cells and NK cells, without concomitant stimulation of regulatory T cells (6–10). Current attention is focused on drug development approaches in which enhanced immune stimulation is achieved by using checkpoint inhibitor antibodies (e.g., anti-PD-1 or anti-PD-L1) as cytokine fusion partners. The resulting cis-activity has been shown to reactivate and expand exhausted effector CD8+ T cells and promoted memory formation, translating into enhanced antitumor efficacy (11–13). Additional potential to overcome tumor resistance may arise from approaches combining cytokine signaling with costimulatory activity, thereby synergistically enhancing immune responses within the tumor microenvironment. Costimulation via 4-1BBL, the natural ligand of 4-1BB, appears particularly well suited for combination strategies. Expression of 4-1BB is induced on TCR-activated T cells and NK cells and serves as an activation marker and a therapeutic target within the tumor microenvironment (14, 15). Costimulation via 4-1BB has been shown to enhance proliferation, effector functions and survival, leading to stronger and more sustained antitumor effects (16). Hence, numerous antibody-based 4-1BB agonists are currently under development (17). Notably, tumor-directed antibody fusion proteins presenting 4-1BBL in trans have shown to deliver costimulatory activity in a targeting-dependent manner, effectively enhancing local antitumor responses in combination with bispecific T-cell engagers (18–20). Antibody-4-1BBL fusion proteins targeting FAP (RO7122290) and CD19 (RO7227166) are being investigated in combination with TCEs directed against CEA (cibisatamab) and CD20 (glofitamab), respectively, in clinical phase I/II studies (NCT04826003, NCT04077723).

Studies on adoptive immune cell transfer support the benefit of combining 4-1BBL-mediated costimulation with IL-15+/- IL-15Rα or IL-21 signaling. Co-expression of these molecules on the surface of K562 feeder cells efficiently expanded NK cells ex vivo, resulting in enhanced cytotoxicity *in vitro* and improved antitumor effects in mouse models (21–23). Similarly, genetically engineered red blood cells co-expressing 4-1BBL and IL-15 fused to IL-15Rα induced T cell and NK cell activation and proliferation, enhanced immune cell infiltration, and promoted antitumor activity in mice (24). Furthermore, CAR-T or CAR-NK cells engineered with CAR-constructs containing the 4-1BB signaling domain and IL-15 and/or IL-21 co-expression demonstrated improved persistence, cytotoxicity, and antitumor efficacy in preclinical models (25, 26). In addition, co-expression of IL-15 in GPC3-specific CAR-T cells increased expansion, intratumoral survival, and antitumor activity in cancer patients (27). These findings highlight the therapeutic potential of these particular cytokine/ligand combinations, which could be further explored through alternative strategies such as trifunctional antibody fusion proteins.

We have previously reported the generation of trifunctional antibody fusion proteins composed of an FAP-specific scFv, human IL-15 fused to an IL-15Rα fragment (RD), and the ectodomain of human 4-1BBL or other costimulatory TNFSF members (28, 29). Proof-of-concept studies demonstrated targeting-dependent cooperative immune stimulation *in vitro*. Furthermore, antitumor activity was shown with mouse-compatible versions in a murine model. These findings support further development of this approach and provide the rationale for the present study.

Here, we describe a novel format of trifunctional fusion protein combining IL-15 fused to an IL-15Rα fragment (RD) and single-chain 4-1BBL by using an antibody Fab unit. In addition, we extend this strategy for the first time to the combination of IL-21 and sc4-1BBL and highlight functional differences between these molecules analyzing immune cell responses of activation, proliferation IFN-γ release and granzyme B expression. Furthermore, combinatorial potential enhancing the efficacy of tumor cell killing mediated by TCE and CTX is assessed.

## 2 Materials and methods

### 2.1 Materials

Antibodies and other reagents were purchased from BioLegend (anti-CD3 PE, 317308; anti-CD8 Pacific Blue, 344718; anti-CD56 APC, 362504; anti-CD69 FITC, 310904; anti-CD56 PacificBlue, 362519; FoxP3 Fix/Perm buffer,421401; FoxP3 Perm buffer, 421402), Miltenyi Biotech (anti-His-PE, 130-120-718; anti-CD8 PEVio770. 130-110-680; anti-CD4 PEVio770, 130-113-789; anti-CD137 APC, 130-110-901; anti-Granzyme B FITC, 130-101-355; CytoStim™, 130-092-173; 7-AAD, 130-111-568) and Merck (Cetuximab). Human IFN-γ (DY285) DuoSet^®^ ELISA kit was purchased from R&D Systems. eIg-based TCE e11-1–1 was made available by O. Seifert (AG Kontermann, University of Stuttgart). 4-1BB-Fc was produced in-house. B16-FAP cells (transfectants with human FAP) (K. Pfizenmaier, University of Stuttgart) were cultured in RPMI 1640 (Life Technologies, 11875), 5% FBS (PAN Biotech, P30-3309), supplemented with 200 µg/ml zeocin (Thermo Fisher Scientific, J67140.XF). IKP-CAF-001 are immortalized primary CAFs from a NSCLC adenocarcinoma modified by Moshe Oren’s Lab (Weizmann Institute of Science, Israel) (30). Cells were cultured in DMEM (Life Technologies, 41965), 10% FBS and 1% Penicillin-Streptomycin (Life Technologies, 15140122). HEK293-6E cells were cultured in Freestyle F17-Medium (Thermo Fisher Scientific, A1383501) supplemented with 4 mM GlutaMAX-I (Thermo Fisher Scientific, 35050038), 0,1% Kolliphore P188 (Merck, K4894). Human peripheral blood mononuclear cells (PBMC) were isolated from buffy coat of healthy donors (Klinikum Stuttgart/Institut für Klinische Transfusionsmedizin und Immungenetik Ulm gemeinnützige GmbH, Germany) by Ficoll density gradient centrifugation (Lymphocyte Separation Medium 1077, Promocell, C-44010) and cultivated in RPMI 1640, 10% FBS. Mitomycin C (M0503) was purchased from Merck.

### 2.2 Generation of antibody-cytokine fusion proteins

FAP-directed antibody fusion proteins were generated based on Fab fragments of IgGhu36 (31). Human IL-21 (UniProtKB Q9HBE4) and RD_IL-15 (aa 31–107 IL-15Rα UniProtKB Q13261, IL-15 UniProtKB P40933) (32) were fused to the C-terminus of the CH1 domain, respectively. A hexahistidyl-tag was placed at the end of the heavy chain. sc4-1BBL, composed of three ectodomains (aa 71-254, UniProtKB P41273) connected by (GGGGS)_4_ linkers into a single chain (29), was fused to the C-terminus of the antibody CL domain. Antibody fragments were connected to the cytokine/ligand component via a glycine/serine-based linker of 10 amino acids. Bi- and trifunctional antibody fusion proteins were assembled by co-expression of the respective heavy/light chain combinations. Recombinant proteins were produced by transient transfection in HEK-293-6E cells (NRC Biotechnology Research Institute, Canada) according to the standard protocol of the cell line provider, followed by purification via immobilized metal ion affinity chromatography (33) or protein A affinity chromatography (TCE) (34). Where possible, the monomeric fraction was enriched by size-exclusion chromatography (Superdex™200 increase, 10/300 GL, GE Healthcare). Proteins were stored at -80 °C and used after no more than one freeze-thaw cycle. Endotoxin levels were not systematically quantified in the present study. However, all protein preparations were produced under standardized endotoxin-minimizing conditions.

### 2.3 Thermostability assay

Thermostability of the fusion proteins was determined by dynamic light scattering with the ZetaSizer Nano ZS (Malvern). Purified protein in PBS was sterile-filtered and exposed to increasing temperatures (35 °C to 85 °C) in 1 °C intervals with 2-minute equilibration steps. The melting point was defined by the starting point of the increase in light scattering.

### 2.4 Binding analysis by flow cytometry

2x10^5^ target cells/well were incubated with the respective fusion protein for 1 h at 4 °C. Bound protein was detected by PE-conjugated anti-hexahistidyl-tag antibody, respectively. Fluorescence was measured by MACSQuant Analyzer10/VYB (Miltenyi Biotech), and data were analyzed using FlowJo (Tree Star). Relative mean fluorescence intensity (MFI) was calculated as (MFI_sample_-(MFI_detection system_-MFI_cells_))/MFI_cells_.

### 2.5 IFN-γ release assay

2x10^4^ B16-FAP cells/well were seeded in 96-well-plates. In parallel, PBMCs were thawed. The next day, B16-FAP cells were arrested with 10 µg/ml mitomycin (2 h, 37 °C), followed by the incubation with the fusion proteins (1 h, RT). After washing, 1:10.000 CytoStim™ (suboptimal concentration) and 2x10^5^ PBMC/well were added. In the untargeted setting, PBMCs were directly incubated with the fusion proteins. After 2 days of culture, the supernatant was harvested, and IFN-γ concentration was determined via sandwich ELISA (DuoSet ELISA kit) according to the manufacturer’s instructions.

### 2.6 Granzyme B expression assay

2x10^4^ B16-FAP cells/well were seeded in 96-well-plates. In parallel, PBMCs were thawed. The next day, B16-FAP cells were arrested with 10 µg/ml mitomycin (2 h, 37 °C), followed by the incubation with the fusion proteins (1 h, RT). After washing, 1:10.000 CytoStim™ (suboptimal concentration) and 2x10^5^ PBMCs/well were added. In the untargeted setting, PBMCs were directly incubated with the fusion proteins. After 4 days of culture, cells were fixed (20 min, RT, FoxP3 Fix/Perm buffer, permeabilized (15 min, RT, FoxP3 Perm buffer), and stained with anti- CD3 PE, anti-CD8 PEVio770, and anti-Granzyme B FITC antibodies. Cells were measured by flow cytometry (MACSQuant Analyzer10/VYB), and data were analyzed using FlowJo (Tree Star).

### 2.7 Live-cell imaging (Incucyte)

2.5x10^3^ IKP-CAF cells/well were seeded in ultra-low attachment 96-well-plates. After 2 days, spheroids had been formed and antibody fusion proteins and resting PBMCs (thawed the day before) were added. Images were taken by the Incucyte every 3 hours for 6 days using the Incucyte Single Spheroid software module. Spheroid plus PBMC object boundary was identified in the Brightfield channel. Tumor cell killing was measured by green integrated fluorescence decline normalized to the 3h value and confirmed by 7-AAD cell death staining (Incucyte Software). At day 6, cells were resuspended, immune cells labeled (anti-CD3 PE, anti-CD8 Pacific Blue, anti-CD56 APC, anti-CD69 FITC) and analyzed by flow cytometry (MACSQuant Analyzer10). Cell activation by CD69 expression and proliferation by cell count were determined using FlowJo (Tree Star). The gating strategy is shown in **Supplementary Figure 1**.

### 2.8 Statistical analysis

Unless otherwise stated, all data are represented as mean ± S.D. of three independent experiments. A block shift correction was applied to account for day-to-day variability in the assay. Block shift correction was performed according to the formula: X’_n_ = X_n_ – (Y_n_ – Y), with X’_n_ being the corrected value of X from the experiment n, Y the average of the X values from all experiments performed, and Y_n_ the average of the duplicate values of X from experiment n. Statistical significance was determined using one-way ANOVA followed by Tukey’s post-test (Graphpad Prism). *P* values below 0.05 were considered statistically significant (*** *P* < 0.001, ** *P* < 0.01, * *P* < 0.05).

## 3 Results

### 3.1 Generation of trifunctional antibody fusion proteins in Fab format combining 4-1BBL with RD_IL-15 or IL-21

We generated trifunctional antibody-cytokine fusion proteins composed of an antibody moiety directed against the tumor stroma antigen fibroblast activation protein (FAP), the costimulatory ligand 4-1BBL from the TNF superfamily, and either the cytokine IL-15 fused to an IL-15Rα fragment (RD) or IL-21 from the common γ-chain receptor family. In this design, the antibody is present as Fab fragment, fused at the C-terminus of the light chain to single-chain 4-1BBL and at the C-terminus of the heavy chain to the respective cytokine, followed by a hexahistidine tag (**Figure 1A**). In addition, corresponding bifunctional control antibody fusion proteins containing only one payload (either ligand or cytokine) were generated. The fusion proteins were transiently expressed in HEK293-6E cells and purified via immobilized metal affinity chromatography (IMAC). Average yields ranged between 1.6 and 4.5 g/L (**Supplementary Table 1**). The thermostability of IL-15_RD_Fab_sc4-1BBL (67 °C) was higher than that of IL-21_Fab_sc4-1BBL (61 °C), as determined by dynamic light scattering (**Figure 1B**). Serum stability of IL-15_RD_Fab_sc4-1BBL was also higher than that of IL-21_Fab_sc4-1BBL, with 88% versus 64% of the protein remaining intact after 6 days of incubation at 37 °C (**Supplementary Figure 2**). SDS-PAGE analysis revealed bands consistent with the expected individual chains and cytokine glycosylation, and corroborated Fab-mediated heterodimer formation (**Supplementary Figure 3A**). Size exclusion chromatography analysis showed single peaks for the IL-21-containing fusion proteins, whereas the IL-15 containing proteins exhibited a major monomer peak and a minor oligomer peak, predominantly dimers, with the oligomeric fraction accounting for approximately 30% of the bifunctional and 23% of the trifunctional fusion protein. The 4-1BBL-only fusion protein similarly showed a minor oligomer peak, predominantly dimers, accounting for approximately 26% of the protein (**Supplementary Figure 3B**; **Supplementary Table 2**). Binding analysis to B16-FAP cells confirmed correct assembly and demonstrated similar targeting properties, with EC_50_ values of 3–8 nM, and trifunctional antibody fusion proteins slightly enhanced in comparison to the bifunctional variants. Specificity was shown by the absence of binding to B16 wild-type control cells (**Figure 1C**; **Supplementary Table 1**). In addition, binding of the 4-1BB-Fc receptor to the sc4-1BBL moiety of the trifunctional fusion proteins targeted to B16-FAP cells was demonstrated by flow cytometry (**Supplementary Figure 4A**) and confocal microscopy (**Supplementary Figure 4B**), indicating accessible presentation for receptor binding and spatial distribution of sc4-1BBL on the target cell surface. Expression of 4-1BB on activated T and NK cells after stimulation with CytoStim™ a T-cell engager (EGFRxCD3) (TCE) or Cetuximab (CTX) was determined by flow cytometry after 3 days of incubation in co-cultures of EGFR-expressing cancer-associated fibroblasts and PBMCs. CytoStim™ and the TCE induced 4-1BB expression on T cells and indirectly increased its expression on NK cells, whereas Cetuximab enhanced 4-1BB expression on NK cells only (**Supplementary Figure 5**).

**Figure 1:**
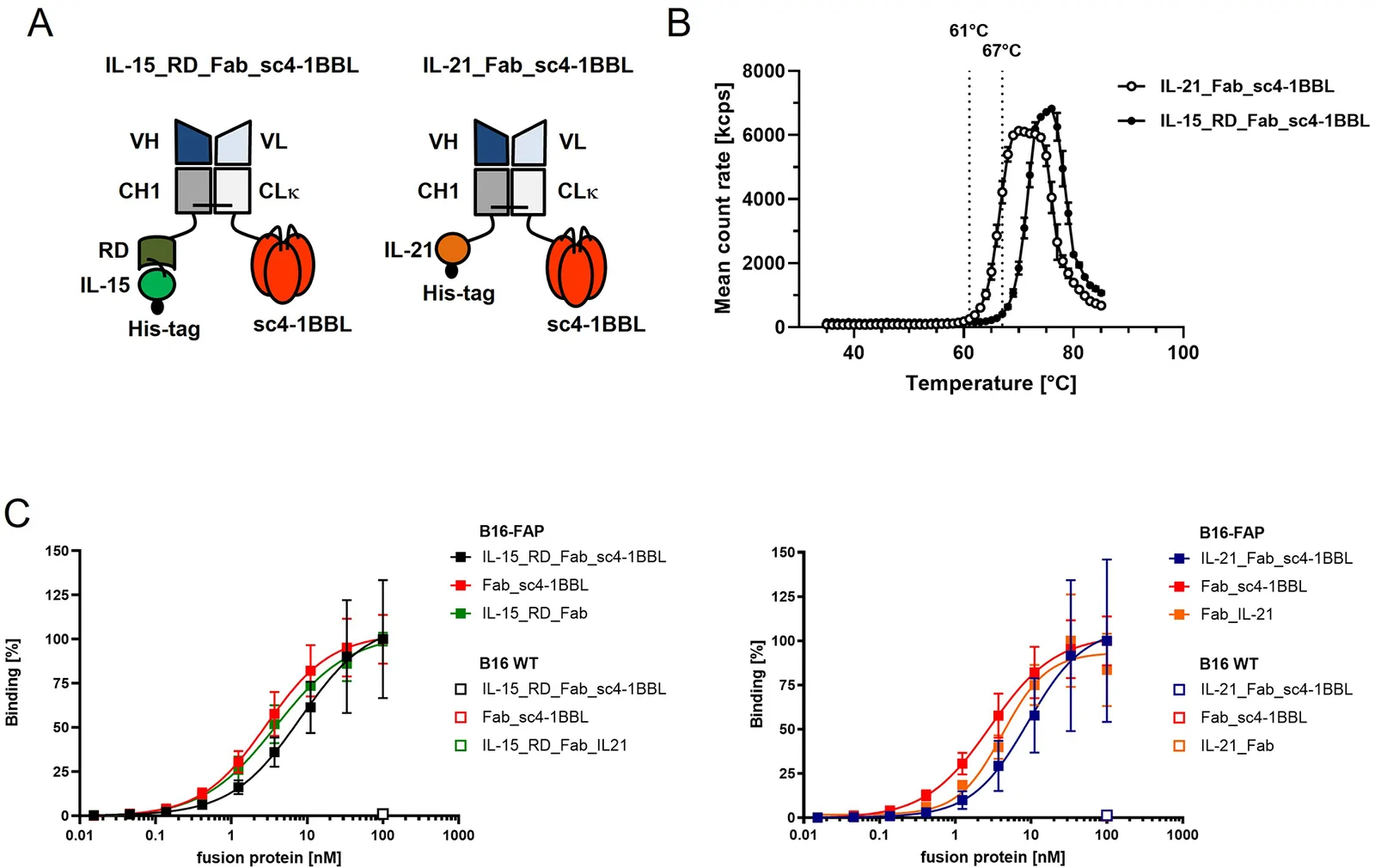
Trifunctional Fab-based fusion proteins combining sc4-1BBL with RD_IL-15 or IL-21. **(A)** Schema of the recombinant proteins. V_H/L_, variable region of the heavy/light antibody chain; CH1, constant domain 1 of γ1 heavy chain; CLk, constant domain of kappa light chain; RD, receptor domain of IL15Rα (aa 31-107); black dot, hexahistidine-tag **(B)** Thermostability analysis of the fusion proteins by dynamic light scattering with the ZetaSizer Nano ZS (Malvern). **(C)** Binding analysis of fusion proteins to B16-FAP cells by flow cytometry. Bound fusion protein was detected by anti-hexahistidine-tag-PE antibody. Graphics show mean ± SD, n=3 (B16-FAP), n=2 (B16-WT), independent experiments.

### 3.2 Immune stimulatory activity of trifunctional antibody fusion proteins in targeted and untargeted form

The activity of the ligand/cytokine payload was assessed with the antibody-fusion proteins in targeted and untargeted form. In the targeted setting, PBMCs were cocultured with B16-FAP cells presenting antibody fusion proteins bound to FAP on the cell surface. In the untargeted setting, PBMCs were incubated with the antibody fusion proteins in solution. Immune cell stimulation of PBMCs activated with a suboptimal concentration of CytoStim™ (T cell superagonist) was assessed after 2 and 4 days by measuring IFN-γ release and granzyme B expression, respectively.

Untargeted, both trifunctional fusion proteins were active and enhanced IFN-γ release to a similar extent as the corresponding bifunctional antibody-cytokine fusion proteins, either alone or in combination with the bifunctional antibody-4-1BBL fusion protein. As expected, no significant effect was observed for untargeted Fab_sc4-1BBL alone (**Figure 2A**). These findings suggest that the activity of the trifunctional molecule was primarily derived from the cytokine payload, with no detectable additional contribution from the costimulatory ligand in solution, whereby IL-15 was more effective than IL-21. In contrast, in the targeted form, the activity of each trifunctional fusion protein was clearly higher than that of the corresponding bifunctional fusion proteins, either alone or in combination (**Figure 2B**). While the individual activities of both payloads were comparable, the maximal combinatorial enhancement effect of the bifunctional molecules was approximately 1.8-fold for IL-15/4-1BBL and 3-fold for IL-21/4-1BBL. In comparison, the trifunctional molecules showed stronger effects, reaching approximately 3-fold (IL-15/4-1BBL) and 7.5-fold (IL-21/4-1BBL). To corroborate dependency on antibody-mediated targeting, trifunctional control fusion proteins directed against the unrelated target EpCAM, which is not expressed on B16-FAP cells, were tested under the same conditions. No enhancement effects were observed (**Supplementary Figure 6**). Altogether, these findings suggest that in the targeted trifunctional molecule the presence of the costimulatory ligand contributes to IFN-γ induction, with the combination containing IL-21 appearing to be particularly effective.

**Figure 2:**
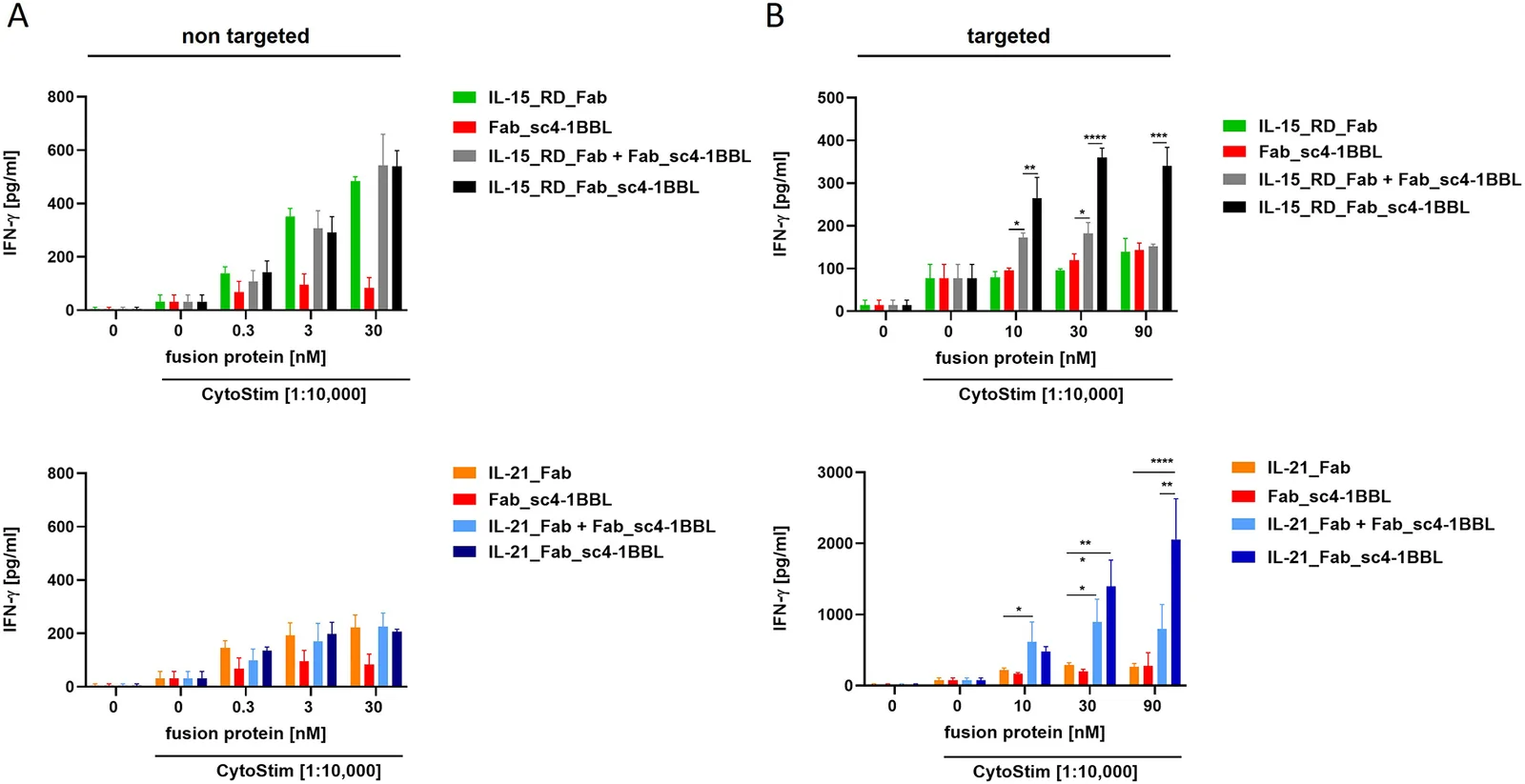
Targeted trifunctional fusion proteins efficiently enhance IFN-γ release. Fusion proteins were incubated **(A)** untargeted or **(B)** targeted to B16-FAP cells (preincubated with the indicated concentration and then washed to remove unbound fusion protein) with PBMCs in presence of suboptimal concentration of CytoStim™. After 2 days, IFN-γ was quantified in the supernatant by sandwich ELISA. Graphics show mean ± SD, n=3, independent experiments, using PBMCs from the same donor and fusion proteins from batches #1. One-way ANOVA, Tukey’s post-test *p<0.05; **p<0.01; ***p<0.001; ****p<0.0001.

Analysis of granzyme B expression in CD8+ T cells in the untargeted setting revealed a strong enhancing effect of the bifunctional cytokine fusion proteins (IL-15 or IL-21), whereas no measurable effect was observed for the bifunctional 4-1BBL fusion protein alone. Their combination did not result in further relevant enhancement. Notably, compared to the corresponding cytokine-only bifunctional fusion proteins, the effect of the trifunctional IL-15/4-1BBL molecule was reduced (0.2-0.4-fold), suggesting a potential sterically hindering effect of the ligand in solution, whereas the trifunctional IL-21/4-1BBL construct showed an increased effect (2.2-fold), consistent with a potential combined activity of the two payloads (**Figure 3A**). In the targeted setting, all individual payloads were active, with the bifunctional 4-1BBL molecule generally showing a weaker effect than the cytokine-containing bifunctional molecules and no combinatorial effect between the bifunctional constructs was observed. The effect of the trifunctional IL-15/4-1BBL fusion protein was comparable to, or only slightly higher than that of the bifunctional IL-15 construct, whereas the trifunctional IL-21/4-1BBL construct showed a 1.5-fold enhancement compared with the bifunctional IL-21 fusion protein (**Figure 3B**). Thus, the combinatorial enhancement of cytotoxic potential in trifunctional constructs was most pronounced for IL-21/4-1BBL under both targeted and untargeted conditions, whereas the benefit of IL-15/4-1BBL was minimal in the targeted setting and appeared to be reduced in the untargeted setting.

**Figure 3:**
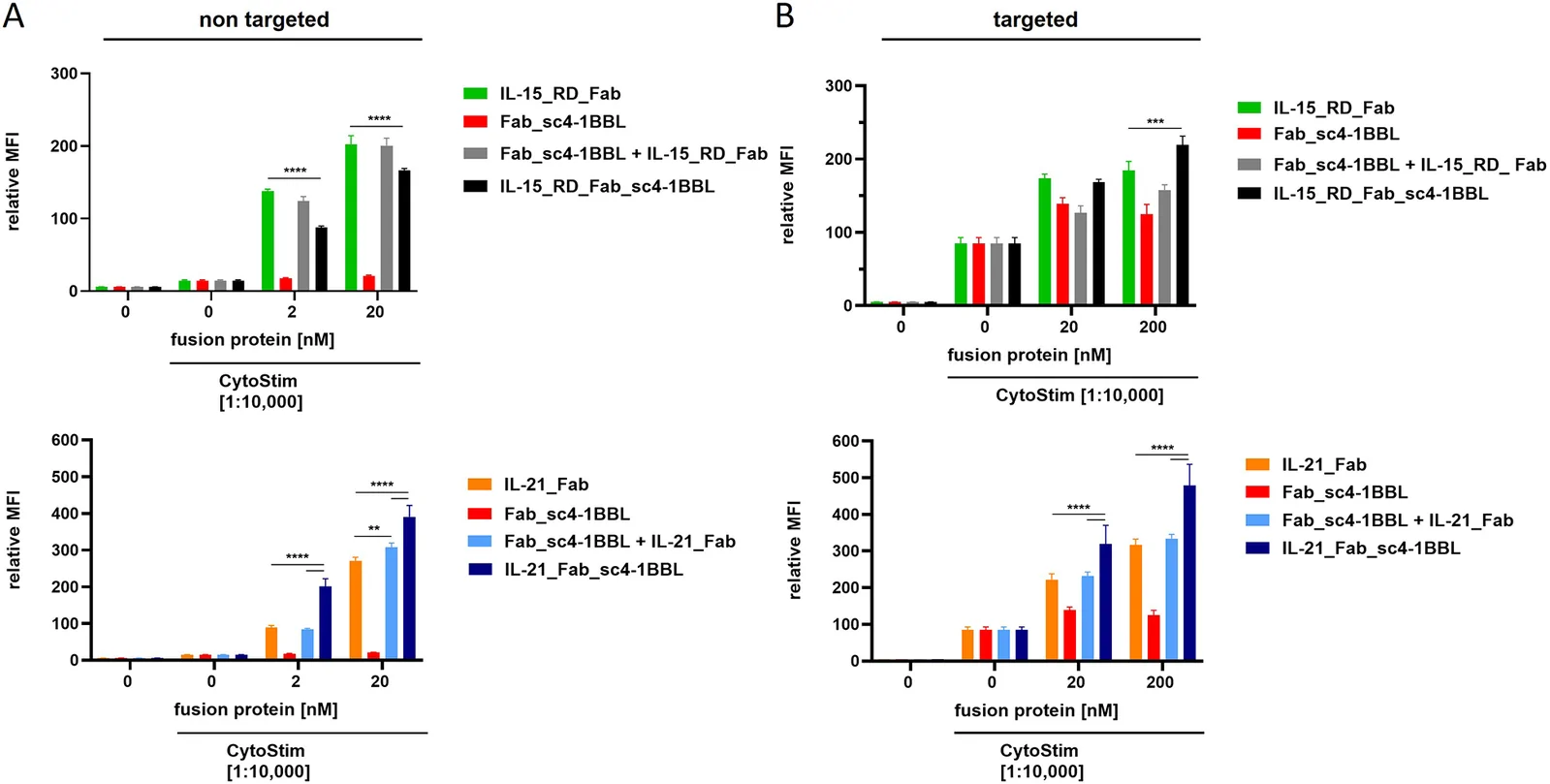
Trifunctional fusion proteins enhance cytotoxic potential differently. Fusion proteins were incubated **(A)** untargeted or **(B)** targeted to B16-FAP cells (preincubated with the indicated concentration and then washed to remove unbound fusion protein) with PBMCs in presence of suboptimal concentration of CytoStim™. After 4 days, cells were fixed, permeabilized and intracellularly stained with anti-CD3, anti-CD8 and anti-granzyme B antibodies, followed by flow cytometry analysis. Graphics show mean ± SD, n=3, independent experiments, using PBMCs from the same donor and fusion proteins from batches #2, with blockshift correction, One-way ANOVA, Tukey’s post-test **p<0.01; ***p<0.001; ****p<0.0001.

### 3.3 Trifunctional fusion proteins enhance the antitumor response of a T-cell engager (EGFRXCD3) and cetuximab

Next, influence on target cell killing was assessed in a 3D setting using spheroids of cancer-associated fibroblasts expressing FAP and EGFR (IKP-CAF-001 GFP+), cocultured with resting PBMCs in the presence of 0.4 nM trifunctional antibody-fusion proteins either alone or in combination with an EGFR-directed T cell engager or cetuximab at 0.25 nM. Tumor cell killing was monitored by live-cell imaging over 6 days, followed by analysis of activation marker and cell counts of immune cell populations.

Bi- and trifunctional antibody-fusion proteins carrying the same cytokine payload showed comparable efficacy in enhancing naturally occurring PBMC-mediated cytotoxicity (maximum ~40% killing). Over a period of 4–5 days, IL-15 (up to 100% killing) was more efficient than IL-21 (maximum ~70% killing) (**Figure 4A**). Artificial T cell retargeting by the T cell engager (TCE) was more potent, reaching ~90% killing after 3 days. Combination with either trifunctional fusion protein slightly improved cytotoxicity, achieving ~100% killing after 2 days (**Figure 4B**). In contrast, antibody-dependent cellular cytotoxicity (ADCC) mediated by cetuximab was less potent and efficient (maximum ~70% killing after 4 days) than TCE-mediated killing. However, both potency and efficacy were enhanced by combination with the trifunctional fusion proteins containing IL-15/4-1BBL and IL-21/4-1BBL, resulting in almost 100% killing after 2 and 3 days, respectively (**Figure 4C**). Similar results were obtained with the bifunctional antibody-cytokine fusion proteins, indicating no detectable additional contribution by 4-1BBL (**Supplementary Figures 7**, **8**). Thus, PBMC-mediated target cell killing could be enhanced by both trifunctional antibody-fusion proteins, either as single agents or in combination with low-dose TCE or cetuximab. Overall, the trifunctional IL-15/4-1BBL construct was more potent than the trifunctional IL-21/4-1BBL construct. However, under these conditions, no apparent advantage of the trifunctional over the respective bifunctional cytokine fusion proteins was observed, suggesting that the benefit of incorporating 4-1BBL may be context dependent.

**Figure 4:**
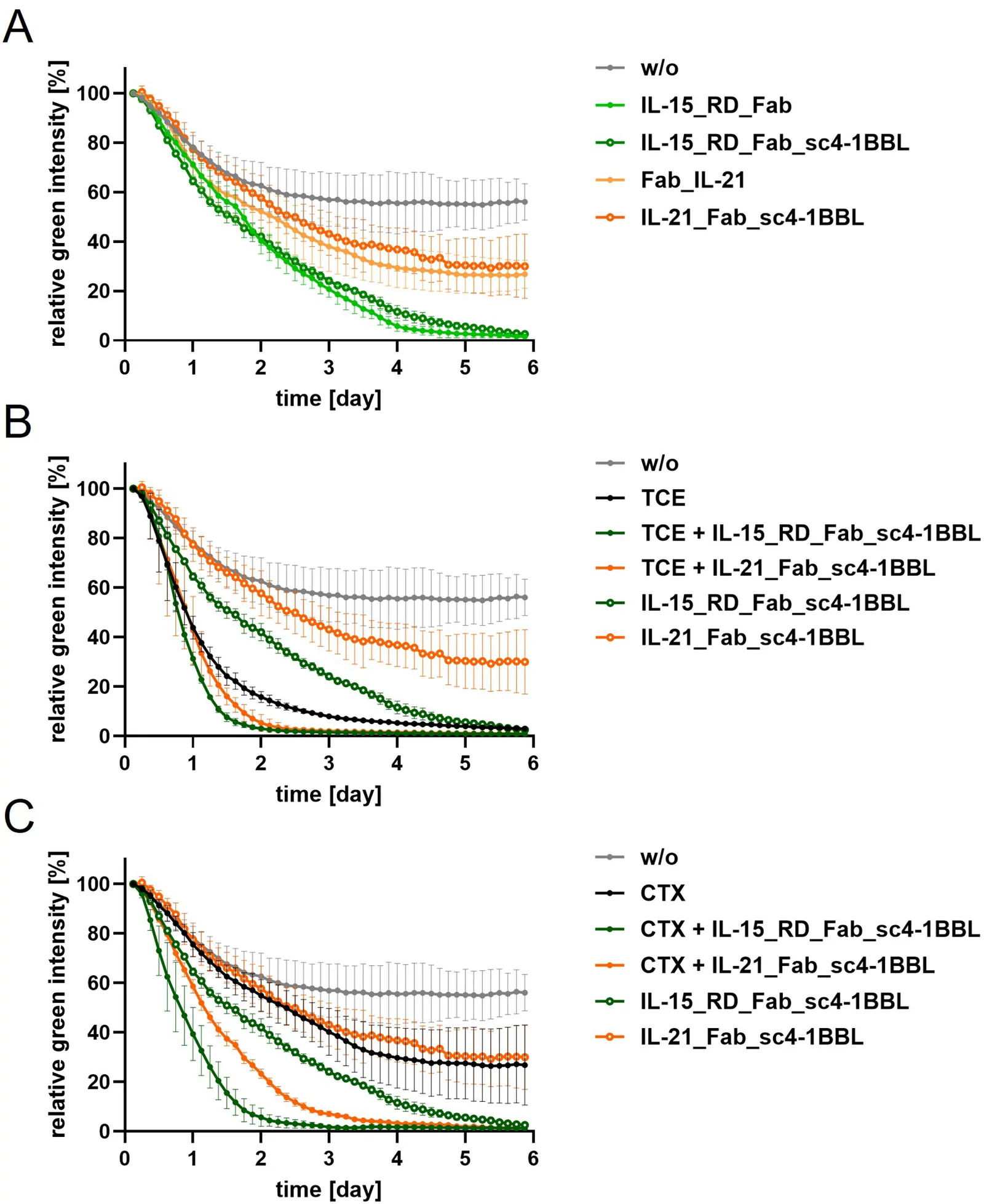
Trifunctional fusion proteins enhance TCE- and Cetuximab-induced target cell killing. IKP-CAF-GFP spheroids were cocultured with resting PBMCs in presence of 0.4 nM fusion protein alone **(A)** and in combination with either 0.25 nM TCE **(B)** or Cetuximab **(C)**. Cytotoxicity was monitored for 6 days via live cell imaging (Incucyte), measuring green fluorescence reduction. Data of spheroids was normalized to the 3 h value. Graphics show mean ± SD, n=3, independent experiments, using PBMCs from the same donor and fusion proteins from batches #3, with blockshift correction.

In accordance with the cytotoxicity results, endpoint analysis of major effector cell populations in terms of activation (**Figure 5A**) and expansion (**Figure 5B**) revealed comparable activity between bi- and trifunctional antibody fusion proteins carrying the same cytokine payload, but pronounced differences between the individual cytokines. IL-15 strongly activated CD8^+^ T cells in the absence (up to 55%) and in the presence of TCE (up to 65%), resulting in approximately 2-fold and 6-fold expansion of the CD8^+^ T-cell population, respectively. In contrast, IL-21 activated CD8^+^ T cells only in combination with TCE (up to 40%), and no expansion effect was observed. As expected, combination with cetuximab did not enhance T-cell stimulation. A similar pattern was observed for CD3+CD56+ NKT-like cells. IL-15 increased activation from approximately 40% to 80%, resulting in strong expansion both in the absence (40-fold) and particularly in the presence of TCE (69-fold). In comparison, IL-21 induced weak activation in presence of TCE (from ~40% to ~60%) and no significant increase in cell number. For NK cells, IL-15 again showed strong activity, increasing activation from ~40% to ~85%. A modest enhancement by IL-21 was most consistently observed in combination with cetuximab (from ~45% to ~60%). Proliferation was observed with both cytokines, resulting in a 4-fold (IL-21) and a 6-fold (IL-15) increase in NK cell numbers, respectively, independent of the combination partner.

**Figure 5:**
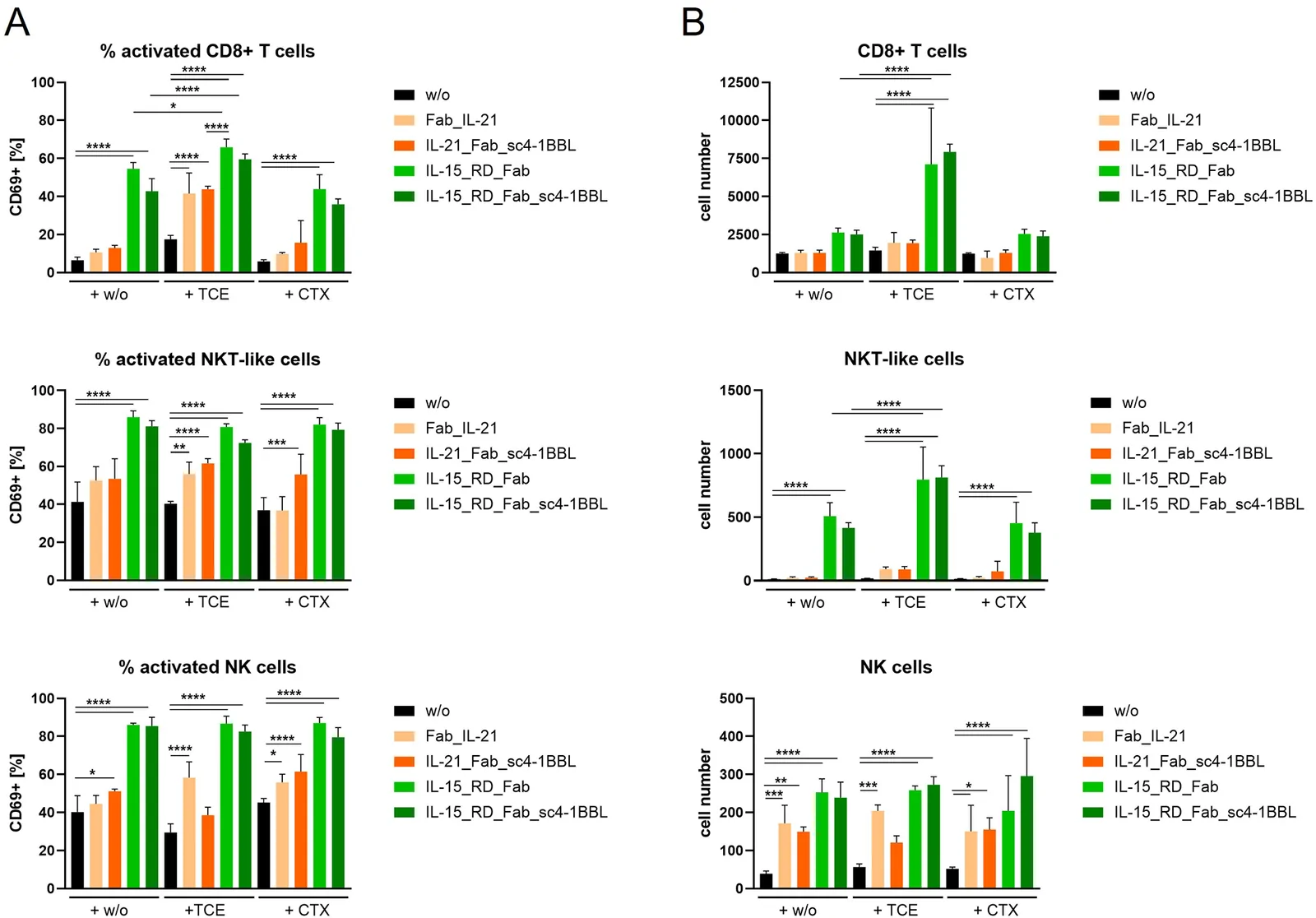
Fusion proteins with IL-15 and IL-21 impact differently activation and proliferation of immune cell effector populations. Fibroblast spheroids were cocultured with resting PBMCs in presence of 0.4 nM fusion protein (batch #3) alone and in combination with either 0.25 nM TCE or Cetuximab. After 6 days, activation and proliferation of CD8+ T cells (CD3+CD8+), NKT-like cells (CD3+CD56+) and NK cells (CD3-CD56+) were determined measuring CD69 expression **(A)** and cell count **(B)** by flow cytometry. Graphics show mean ± SD, n=3, independent experiments, using PBMCs from the same donor and fusion proteins from batches #3, with blockshift correction, One-way ANOVA, Tukey’s post-test *p<0.05; **p<0.01; ***p<0.001; ****p<0.0001.

Thus, consistent with PBMC-mediated target cell killing, antibody fusion proteins containing IL-15 were more effective than those containing IL-21 in activating CD8^+^ T cells, NKT cells, and NK cells. Distinct patterns of activity were observed: IL-15 efficiently induced proliferation of CD8^+^ T cells and NKT-like cells, particularly in combination with TCE, whereas IL-21 did not. Both cytokines, however, promoted NK cell proliferation. Overall, both trifunctional antibody fusion proteins enhanced the cytotoxic effects of the T-cell engager and cetuximab, supporting their potential for combination with T-cell– and NK cell–based immunotherapeutic strategies, respectively.

## 4 Discussion

We report the design of trifunctional antibody fusion proteins for the colocalized delivery of a stimulatory cytokine (IL-15 or IL-21) and a costimulatory ligand (4-1BBL), i.e., molecules with distinct activities and receptor activation requirements. For proper activation of TNF receptor superfamily members such as 4-1BB, receptor clustering is essential (2). Under physiological conditions, this is achieved by trans-presentation of the trimeric ligand as a transmembrane protein, but not by the soluble (shed) ligand. Strategies that mimic cell-surface presentation, such as tumor-directed antibody fusion proteins carrying ligand ectodomains or 4-1BB bispecific antibodies, exploit this conditional activity. The costimulatory nature of this signal further increases the attractiveness of this approach for combinatorial strategies, and several agonists are currently in clinical trials (17). IL-15 and IL-21, in contrast, are cytokines of the common γ-chain receptor family that are active in soluble form. IL-15, however, is physiologically presented in trans via IL-15Rα on the cell surface, resulting in enhanced biological activity. Incorporation of the IL-15Rα-binding domain into recombinant fusion proteins leads to targeting-independent gain of function and is widely used in clinical development (35). Preclinical studies have further demonstrated benefits of targeting via trans-presentation on tumor cells or cis-presentation on exhausted T cells (4, 36). Interestingly, we observed that when a ligand with targeting-dependent activity and a cytokine with targeting-independent activity are combined within a single molecule, their intrinsic properties can be retained, enabling targeting-dependent modulation of their combined activity. In this study, both targeted trifunctional antibody fusion proteins showed strong combinatorial effects of the cytokine and costimulatory ligand, resulting in enhanced IFN-γ release from activated PBMCs. In contrast, untargeted, activity was comparable to that of cytokine-only constructs, or even reduced in the case of (IL-15/4-1BBL), suggesting that target-bound ligand presentation is required for effective combinatorial IFN-γ induction. Similar targeting-dependent differences in IFN-γ release and also T-cell proliferation were previously observed for trifunctional RD_IL-15/4-1BBL variants differing in antibody (scFv) or ligand design (non-covalent trimer versus single-chain trimer), supporting the concept of conditional costimulatory ligand activity across different formats and the generation of partially conditional trifunctional molecules (28, 29). Further studies will have to corroborate the results with additional independent donors and explore the cooperative nature of the payload combinations by receptor blocking and downstream signaling analysis. In addition, further insight into the molecular balance between the individual payloads will be required, as targeting-independent combinatorial enhancement of granzyme B expression was observed for the trifunctional IL-21/4-1BBL fusion protein, suggesting that the extent of conditional activity may depend on the payload combination and functional readout. In terms of design, 4-1BB agonist development has shown that binding properties, valency, and spatial arrangement of binding units are factors critically influencing receptor clustering and activation dynamics (17). In trifunctional formats, complexity increases due to multiple receptor and target interactions as well as downstream signaling pathways, which is expected to affect the balance between trans- and cis-activity. To avoid off-target toxicity, individual molecular optimization will be required, tuning binding properties and spatial configuration to favor synergistic activity in a target-bound state. Considering that studies with bifunctional antibody fusion proteins containing engineered variants of IL-15 and IL-21 have shown that cytokine activity can be partially restore by targeted cell-surface presentation (37, 38), incorporating cytokines with reduced receptor affinity may be an option to reduce sink effects and lower systemic toxicity. Overall, subsequent molecular development will have to carefully address safety considerations when assessing the therapeutic window.

Both trifunctional fusion proteins demonstrated combinatorial potential enhancing the cytotoxicity of EGFR-directed T-cell engager and cetuximab in a CAF-spheroid/PBMC coculture system. Although the spheroid assay demonstrated enhanced target-cell killing, it did not directly establish the underlying mechanism of cell death. Additional mechanistic studies would be required to determine and specify the contribution of perforin/granzyme-mediated apoptosis to target-cell killing. The effect was primarily cytokine-driven, as trifunctional and bifunctional fusion proteins carrying the same cytokine payload exhibited comparable activity. Comparing the stimulatory potential of cytokine payloads, IL-15-containing fusion proteins were more effective than those containing IL-21, consistent with the higher potency of IL-15 in driving activation and expansion of effector cell populations. This aligns with IL-15Rβ upregulation induced by T-cell engager, which enables strong synergy with IL-15/IL-15Rα and promotes CD8^+^ T-cell expansion as a major mechanism of enhanced TCE activity (39). In contrast, IL-21 primarily enhanced the cytotoxic capacity of pre-existing CD8^+^ T cells, consistent with reports showing that IL-21 pretreatment supports TCE activity by modulating multiple steps of cytotoxic effector function (40). Both cytokines were particularly effective enhancing cetuximab-mediated cytotoxicity, which is consistent with the observed expansion of NK cells. This finding confirms the potential of IL-15/IL-15Rαsushi and IL-21 to enhance cetuximab-mediated ADCC, reported to enhance antitumor effects in preclinical studies (41, 42). In our system, antibody fusion proteins were present in solution during coculture, which may have favored cytokine-driven effects at low concentration and masked targeting-dependent contributions of the costimulatory ligand in the trifunctional fusion proteins. This effect may have been further reinforced by the relatively low antigen expression levels of FAP (~10,000 antibody-binding sites per cell) and EGFR (~24,000 antibody-binding sites per cell), which are at the lower end of the range typically considered optimal for targeted strategies (34, 43). These findings suggest that the extent of combined payload activity depends on the functional readout and experimental context.

Mechanistically, activation via T-cell engagers or ADCC is crucial for the combinatorial approach, as it induces upregulation of 4-1BB on T cells and NK cells, respectively, thereby increasing responsiveness to 4-1BBL-mediated costimulation and promoting improved survival and antitumor responses (44, 45). IL-15 could also contribute inducing 4-1BB expression (41, 46, 47). We observed that T-cell engager- and cetuximab-mediated activation at suboptimal concentration was sufficient to induce immune cell retargeting and cytotoxicity but insufficient to expand effector cell populations, highlighting distinct dose requirements for different biological functions. In addition, the rapid kinetic of TCE-mediated cytotoxicity may have limited the contribution of costimulatory signaling, as 4-1BB upregulation typically peaks only after approximately three days (48, 49). While cytokine activity dominates immediately after administration, combined activity with 4-1BBL may become more relevant at later stages *in vivo*, when the ratio of targeted to untargeted molecules increases through the dynamic of protein retention and clearance. By this means, 4-1BBL-mediated costimulation may contribute to long-term functional effects, including metabolic adaptation, memory formation, resistance to exhaustion, and improved immune cell survival (50–53). Such conditions are not captured in the present model and warrant further investigation.

Evidence from adoptive immune cell transfer studies using NK cells (21–23, 54–57), CAR-NK cells (26) and CAR-T cells (25), highlights the potential of locally confined combinations of IL15 and/or IL-21 with 4-1BBL on the cell surface to enhance effector cell persistence and function. Tumor-targeted trifunctional antibody fusion proteins could translate this rationale into a modular recombinant protein format that complements established antibody-based strategies, such as T-cell engagers and monoclonal antibodies. However, the individual and combined activities of the molecular components must be carefully balanced to fully realize their therapeutic potential. Consequently, further mechanistic studies and protein engineering efforts will be required to optimize their molecular design.

## Data Availability

The raw data supporting the conclusions of this article will be made available by the authors, without undue reservation.
