# Supplementary material for: Trifunctional antibody fusion proteins for targeted co-delivery of 4-1BBL with IL-15 or IL-21 to enhance antitumor immune responses
Source: Front Immunol. 2026 Oct 7;17:1903525. doi: 10.3389/fimmu.2026.1903525 (PMC13683726; doi:10.3389/fimmu.2026.1903525)
Supplement: Supplementary file 1 [file DataSheet1.pdf]

Supplementary Material

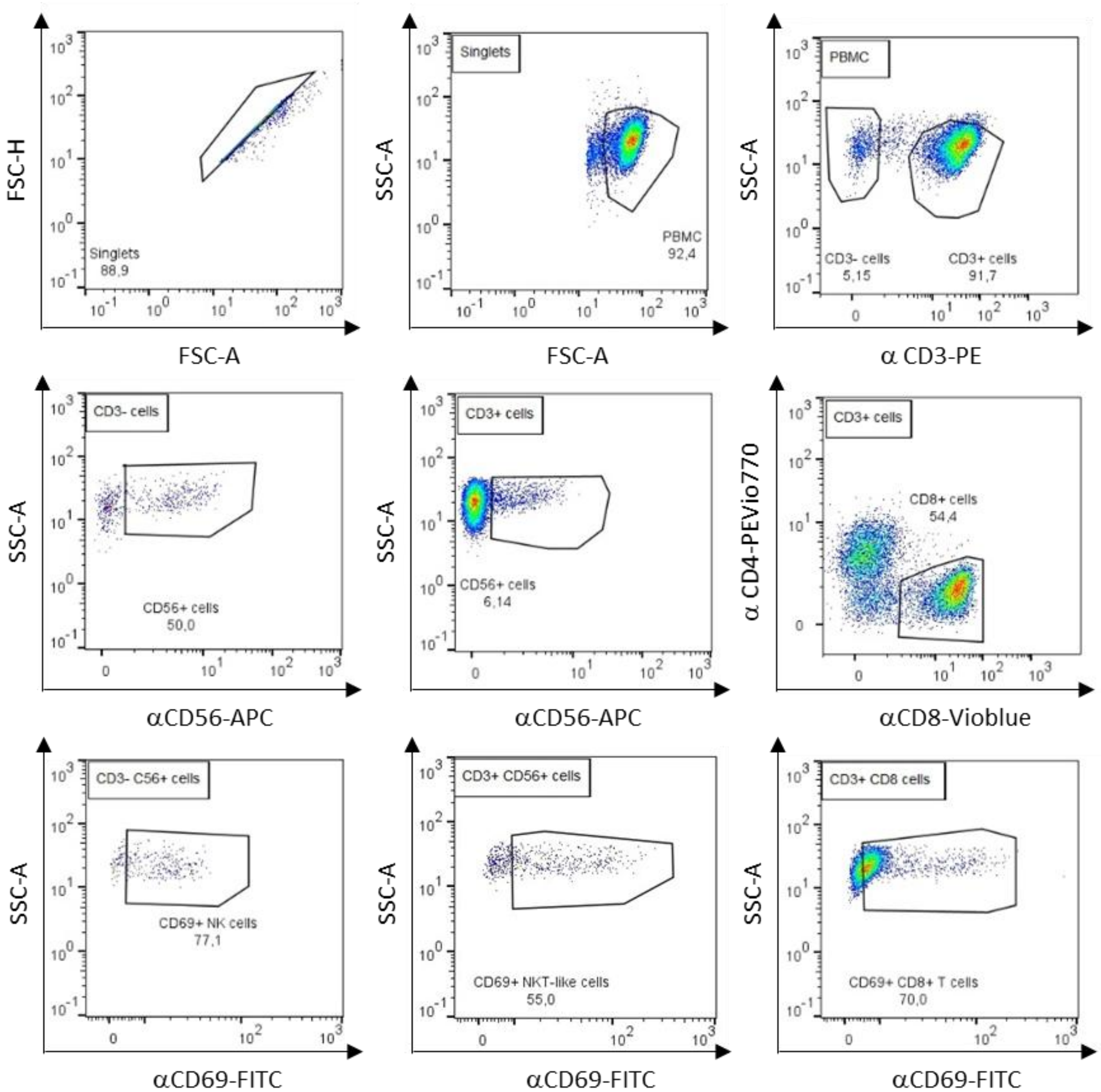

Supplementary Figure 1: Gating strategy for the analysis of activated CD8+ T cells (CD3+CD8+CD69+), NKT-like cells (CD3+CD56+CD69+), and NK cells (CD3−CD56+CD69+). Representative plots are shown for immune cells stimulated with IL-15_RD_Fab_sc4-1BBL + TCE.
