# Supplementary material for: Trifunctional antibody fusion proteins for targeted co-delivery of 4-1BBL with IL-15 or IL-21 to enhance antitumor immune responses
Source: Front Immunol. 2026 Oct 7;17:1903525. doi: 10.3389/fimmu.2026.1903525 (PMC13683726; doi:10.3389/fimmu.2026.1903525)
Supplement: Supplementary file 2 [file DataSheet10.pdf]

Supplementary Material

Table 2 Batch composition of antibody fusion proteins with oligomeric fractions determined by size exclusion chromatography. FPLC: Superdex 200 Increase 10/300 GL, 8,6 µm, 10 mm x 300 mm (GE Healthcare), running buffer DPBS.

<table>
<tr><th>Protein</th><th>Batch</th><th>Monomer</th><th>Dimer</th><th>HMW</th><th>Assay</th></tr>
<tr><td rowspan="3">IL-15_RD_Fab_sc4-1BBL</td><td>#1</td><td>77 %</td><td>18 %</td><td>5 %</td><td>IFN-γ release</td></tr>
<tr><td>#2*</td><td>96 %</td><td colspan="2">4 %</td><td>Granzyme B</td></tr>
<tr><td>#3</td><td>77 %</td><td>17 %</td><td>6 %</td><td>Cytotoxicity</td></tr>
<tr><td rowspan="3">IL-15_RD_Fab</td><td>#1</td><td>70%</td><td>17 %</td><td>13 %</td><td>IFN-γ release</td></tr>
<tr><td>#2</td><td>71%</td><td>18 %</td><td>11 %</td><td>Granzyme B</td></tr>
<tr><td>#3*</td><td>87 %</td><td colspan="2">12 %</td><td>Cytotoxicity</td></tr>
<tr><td rowspan="2">Fab_sc4-1BBL</td><td>#1</td><td>71 %</td><td>22%</td><td>7 %</td><td>IFN-γ release</td></tr>
<tr><td>#2</td><td>78 %</td><td>19 %</td><td>3 %</td><td>Granzyme B</td></tr>
</table>

* Protein batch with monomer enrichment by preparative FPLC (Superdex™200 increase, 10/300 GL, GE Healthcare), running buffer DPBS. SEC analysis by HPLC**:** Superdex™200 increase 8,6 µm, 7,8 mm x 300 mm **(**GE Healthcare), running buffer 50 mM sodium phosphate, 300 mM NaCl, pH 8.0.
