# Supplementary material for: Trifunctional antibody fusion proteins for targeted co-delivery of 4-1BBL with IL-15 or IL-21 to enhance antitumor immune responses
Source: Front Immunol. 2026 Oct 7;17:1903525. doi: 10.3389/fimmu.2026.1903525 (PMC13683726; doi:10.3389/fimmu.2026.1903525)
Supplement: Supplementary file 3 [file DataSheet2.pdf]

Supplementary Material

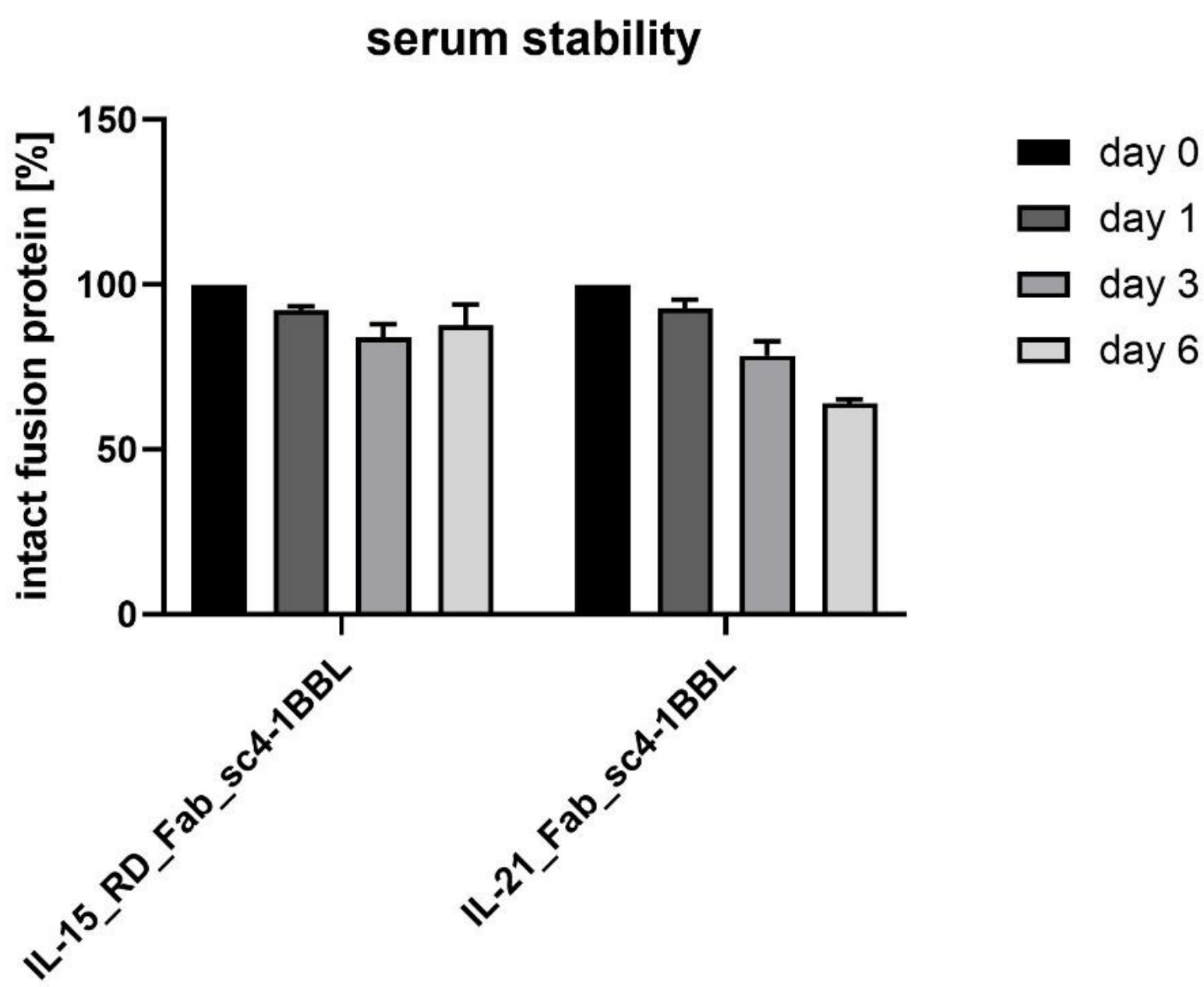

Supplementary Figure 2: Serum stability of trifunctional antibody-fusion proteins. Trifunctional fusion proteins (200 nM) were incubated in 50% human serum at 37°C and aliquots were frozen at the indicated time points. Levels of intact protein were determined by ELISA by measuring binding to immobilized 4-1BB-Fc and detecting bound protein using an HRP-conjugated anti-His-tag antibody. Data were normalized to the binding of the corresponding fusion protein without incubation (day 0). Mean ± SD, n=3.
