# Supplementary material for: Trifunctional antibody fusion proteins for targeted co-delivery of 4-1BBL with IL-15 or IL-21 to enhance antitumor immune responses
Source: Front Immunol. 2026 Oct 7;17:1903525. doi: 10.3389/fimmu.2026.1903525 (PMC13683726; doi:10.3389/fimmu.2026.1903525)
Supplement: Supplementary file 4 [file DataSheet3.pdf]

Supplementary Material

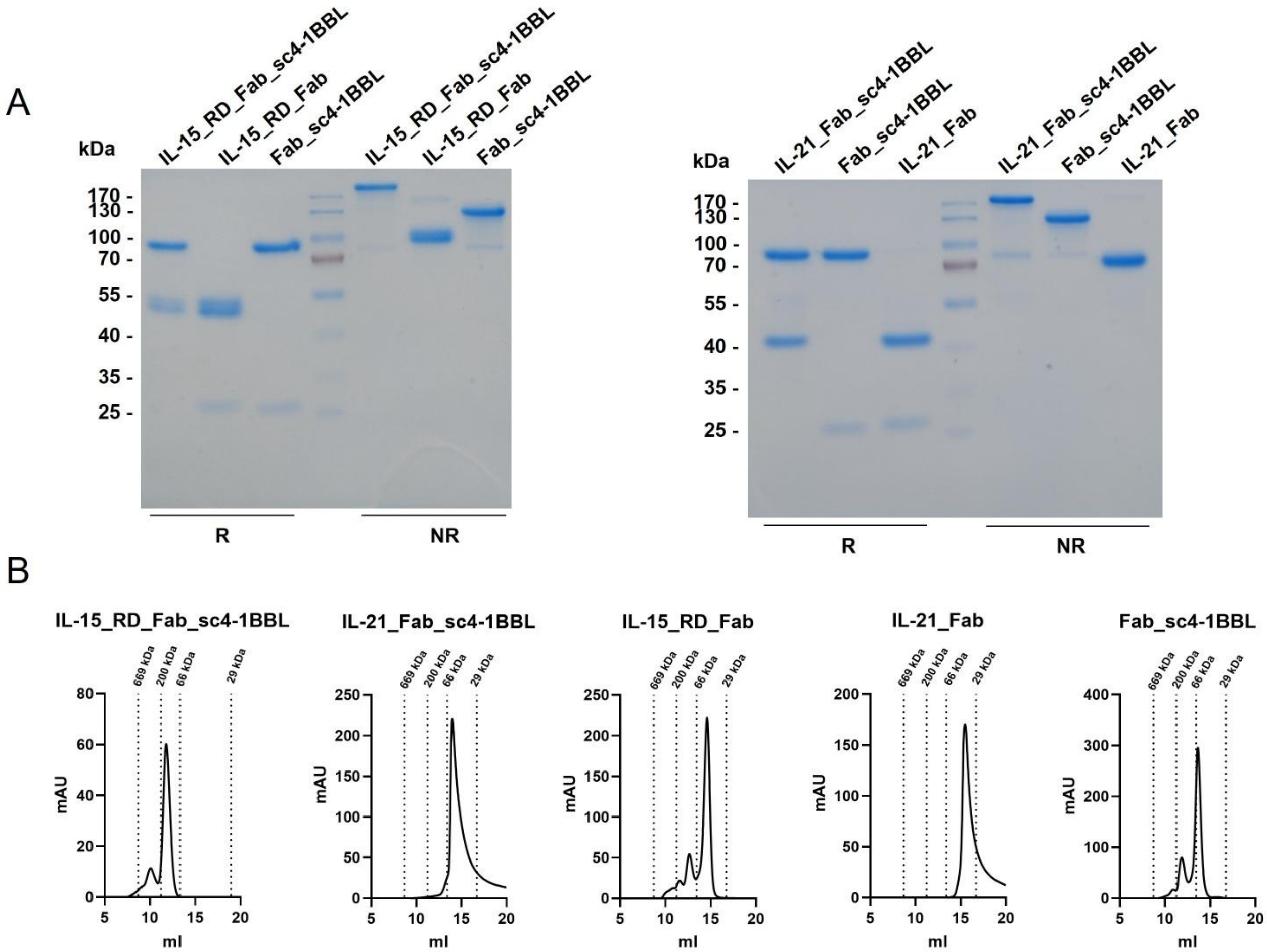

Supplementary Figure 3: Biochemical analysis of bi- and trifunctional fusion proteins after purification via immobilized metal ion affinity chromatography. (A) 12% SDS-PAGE analysis, 4 µg protein/lane, under reducing (R) and non-reducing (NR) conditions. Coomassie staining. (B) SEC-analysis by analytical FPLC, Superdex™200, 10/300 GL, GE Healthcare, buffer DPBS.
