# Supplementary material for: Trifunctional antibody fusion proteins for targeted co-delivery of 4-1BBL with IL-15 or IL-21 to enhance antitumor immune responses
Source: Front Immunol. 2026 Oct 7;17:1903525. doi: 10.3389/fimmu.2026.1903525 (PMC13683726; doi:10.3389/fimmu.2026.1903525)
Supplement: Supplementary file 5 [file DataSheet4.pdf]

Supplementary Material

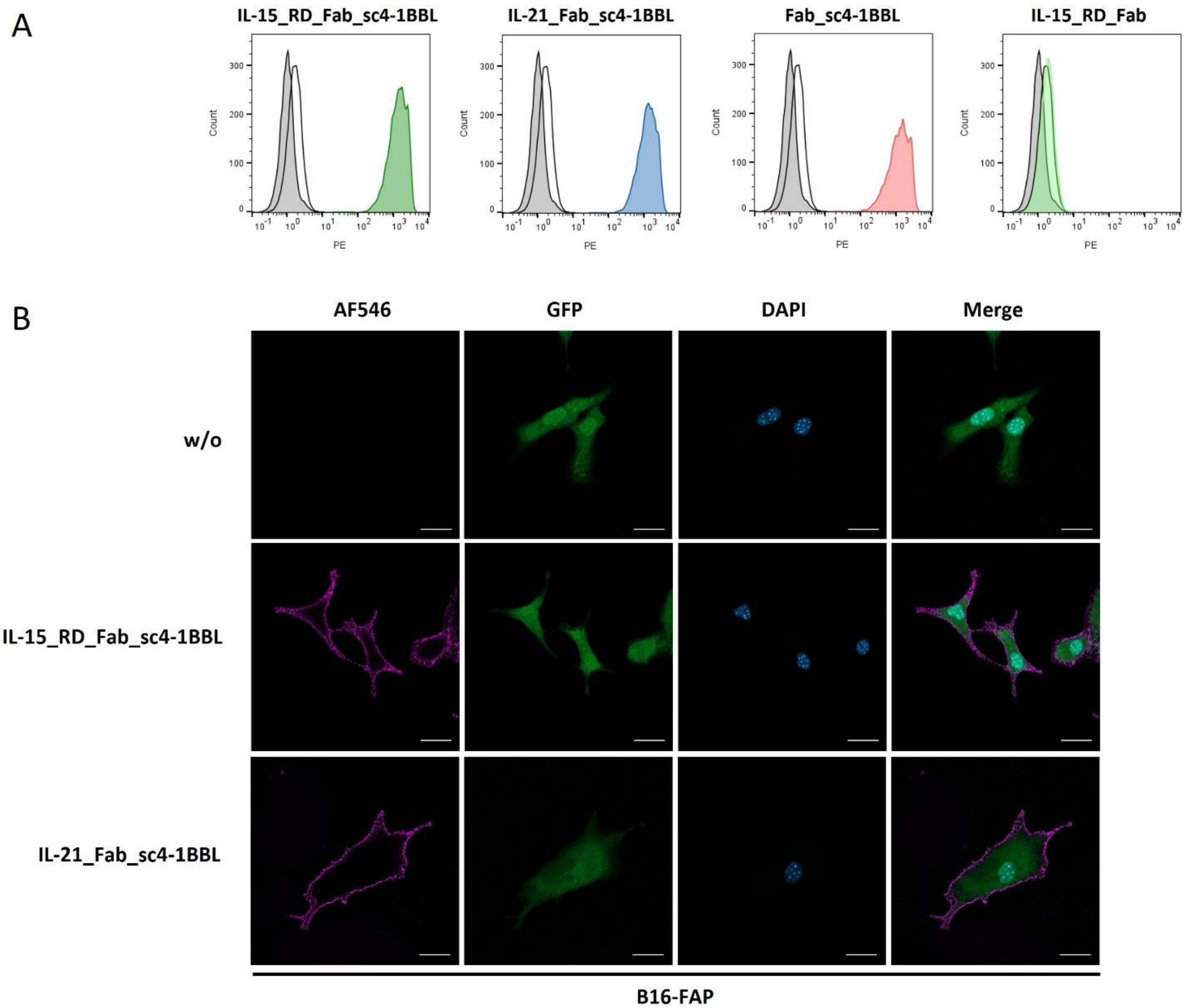

Supplementary Figure 4: 4-1BB-Fc receptor binding to the sc4-1BBL moiety of targeted trifunctional antibody fusion proteins on the cell surface. **(A)** Flow cytometry analysis: $2x10^5$ B16-FAP cells/well were incubated with 100 nM antibody fusion protein for 1 h at 4°C. Bound fusion protein was detected via sc4-1BBL using 4-1BB-Fc (5 µg/ml, 1h, 4°C), followed by a PE-conjugated anti-Fc antibody (Biozol, JIM-109-115-098; 1/500, 1h, 4°C). Fluorescence was measured using a MACSQuant Analyzer10 (Milteny Biotech), and data were analyzed using FlowJo (Tree Star). **(B)** Microscopy analysis: $8x10^4$ B16-FAP (GFP+) cells/well were seeded on Collagen R-coated coverslips (2.5 µg/ml, Serva # 47254). The following day, cells were incubated with 50 nM antibody fusion protein for 30 min on ice. Bound fusion protein was detected via ligand-receptor interaction with 4-1BB-Fc (5 µg/ml, 30 min on ice). After fixation (4% paraformaldehyde, 20 min, RT) and blocking (5% FCS in PBS, 20 min, RT), bound receptor was detected using Alexa Fluor 546-conjugated anti-human IgG antibody

(Invitrogen, A21089; 1/500, 1h, RT). Cells were mounted using ProLong^{TM} Gold Antifade Reagent with DAPI (Life Technologies, P36931). Images were acquired using an LSM 980 Airyscan (Zeiss) equipped with a Plan-Apochromat 63x/1.40 objective, AF546 (Ex/Em:561/560-679 nm), GFP (Ex/Em: 488/489-560 nm), DAPI (Ex/Em: 405/410-604 nm) were used for detection. A single z-layer is shown. Scale bar: 20 µm.
