# Supplementary material for: Trifunctional antibody fusion proteins for targeted co-delivery of 4-1BBL with IL-15 or IL-21 to enhance antitumor immune responses
Source: Front Immunol. 2026 Oct 7;17:1903525. doi: 10.3389/fimmu.2026.1903525 (PMC13683726; doi:10.3389/fimmu.2026.1903525)
Supplement: Supplementary file 6 [file DataSheet5.pdf]

Supplementary Material

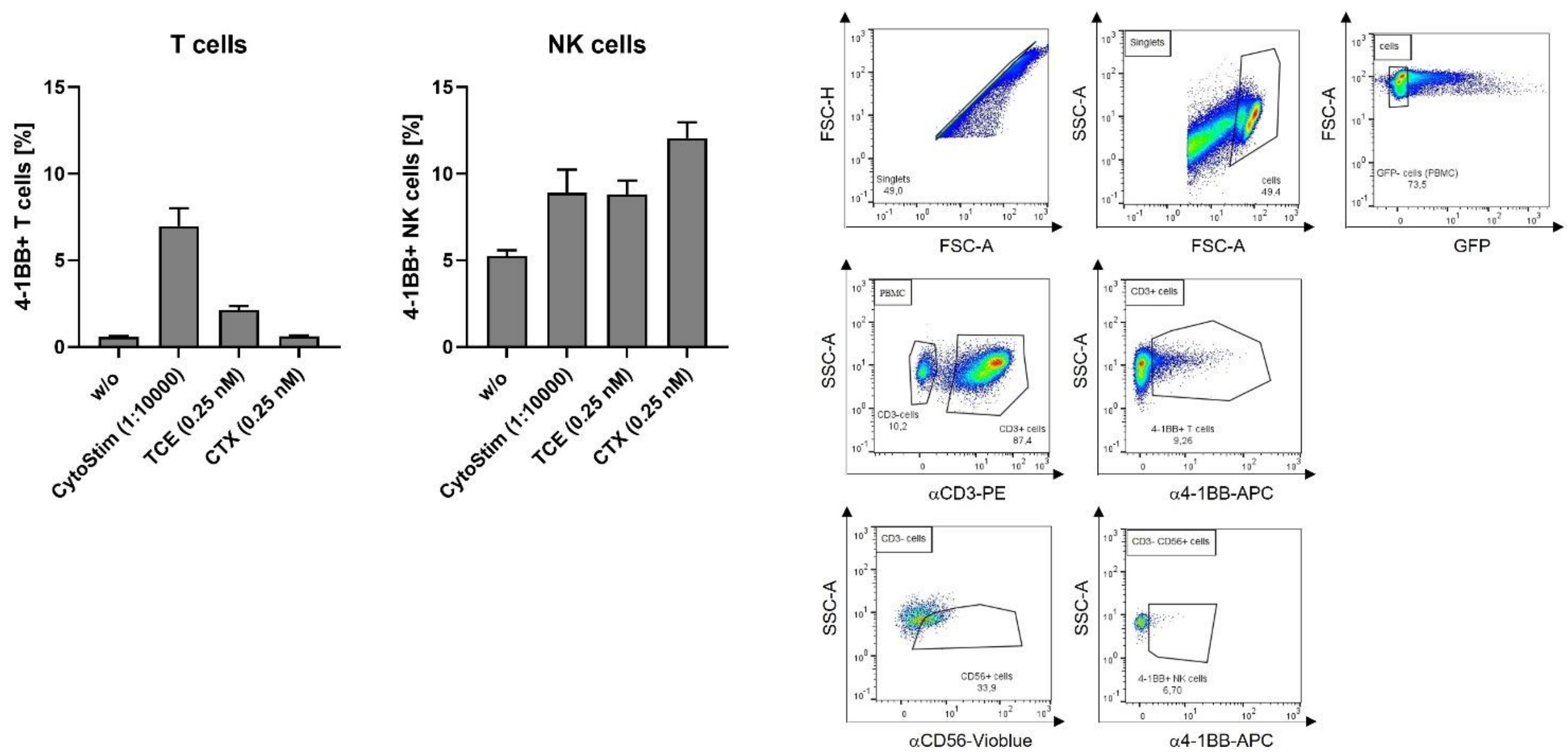

Supplementary Figure 5: 4-1BB expression on stimulated T cells and NK cells. $4x10^4$ IKP-CAF (GFP+) cells/well were seeded. The following day, $2x10^5$ PBMCs/well were added and cocultured in the presence of either CytoStim™ (1:10,000), TCE (0.25 nM) or CTX (0.25 nM) for 3 days. PBMCs from 2 wells were pooled, stained with anti-CD3-PE, anti-CD56-VioBlue and anti-4-1BB-APC antibodies (1h, 4°C) and analyzed by flow cytometry using a MACSQuant Analyzer10 (Milteny Biotech), followed by data analysis using FlowJo (Tree Star). T cells (CD3+CD56-) and NK cells (CD3-CD56+) were identified and 4-1BB+ cells quantified using the indicated gating strategy. Graphic shows mean ± SD of duplicate measurements, n=1.
