# Supplementary material for: Trifunctional antibody fusion proteins for targeted co-delivery of 4-1BBL with IL-15 or IL-21 to enhance antitumor immune responses
Source: Front Immunol. 2026 Oct 7;17:1903525. doi: 10.3389/fimmu.2026.1903525 (PMC13683726; doi:10.3389/fimmu.2026.1903525)
Supplement: Supplementary file 7 [file DataSheet6.pdf]

Supplementary Material

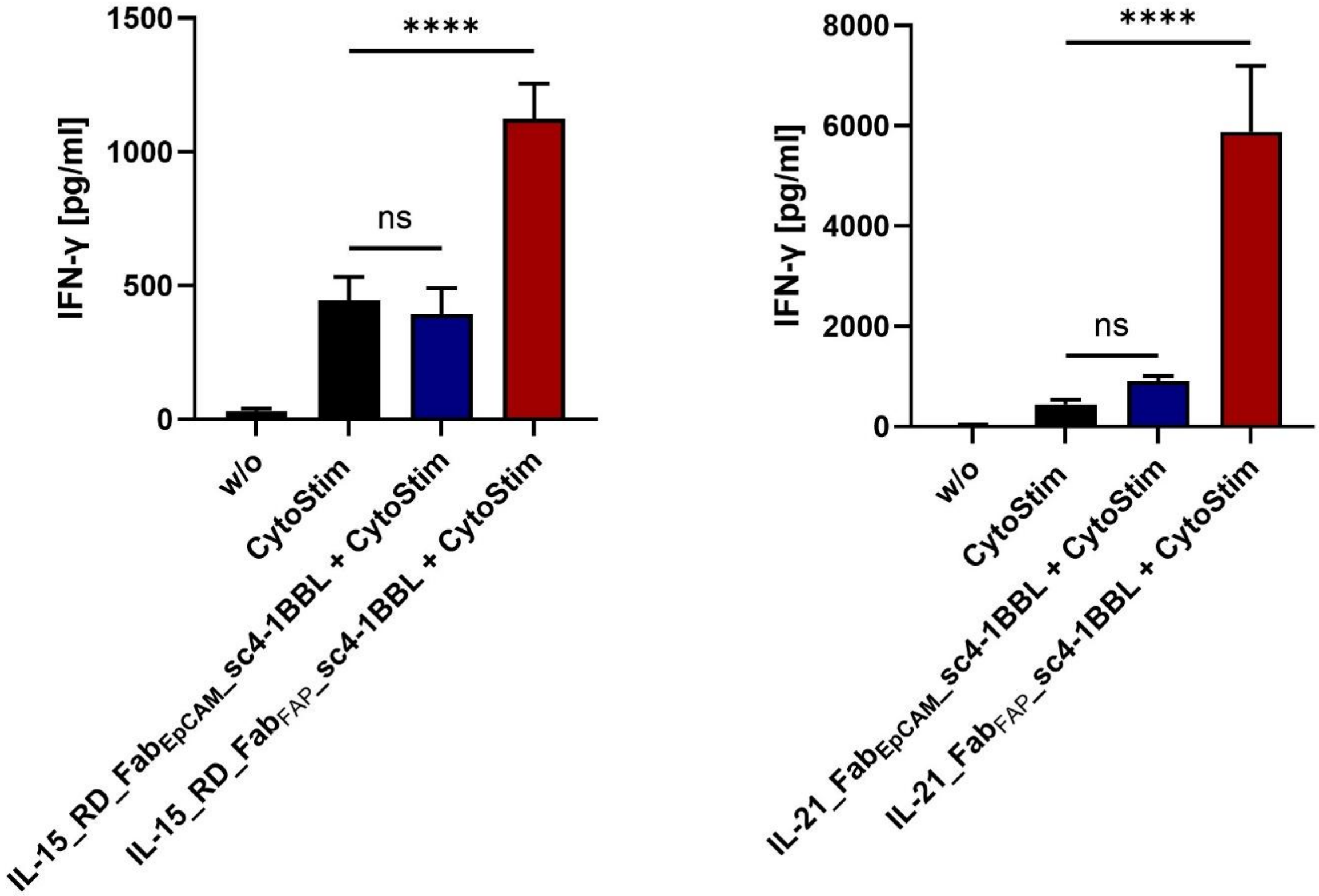

Supplementary Figure 6: Targeted trifunctional fusion proteins enhance IFN-γ release from activated T cells. B16-FAP cells were incubated with target-specific (FAP) antibody fusion proteins or control antibody fusion proteins directed against an unrelated target (EpCAM), which is not expressed on B16-FAP cells. After preincubation with 30 nM fusion protein, unbound fusion protein was removed by washing, followed by the addition of PBMCs in the presence of CytoStim™ (1:10,000). After 2 days, IFN-γ levels in the supernatant were quantified by sandwich ELISA. The graphs show mean ± SD, n=3, independent experiments using PBMC from the same donor. One-way ANOVA with Tukey's post-test. ns, not significant; ****$p<0.0001$.
