# Supplementary material for: Trifunctional antibody fusion proteins for targeted co-delivery of 4-1BBL with IL-15 or IL-21 to enhance antitumor immune responses
Source: Front Immunol. 2026 Oct 7;17:1903525. doi: 10.3389/fimmu.2026.1903525 (PMC13683726; doi:10.3389/fimmu.2026.1903525)
Supplement: Supplementary file 8 [file DataSheet7.pdf]

Supplementary Material

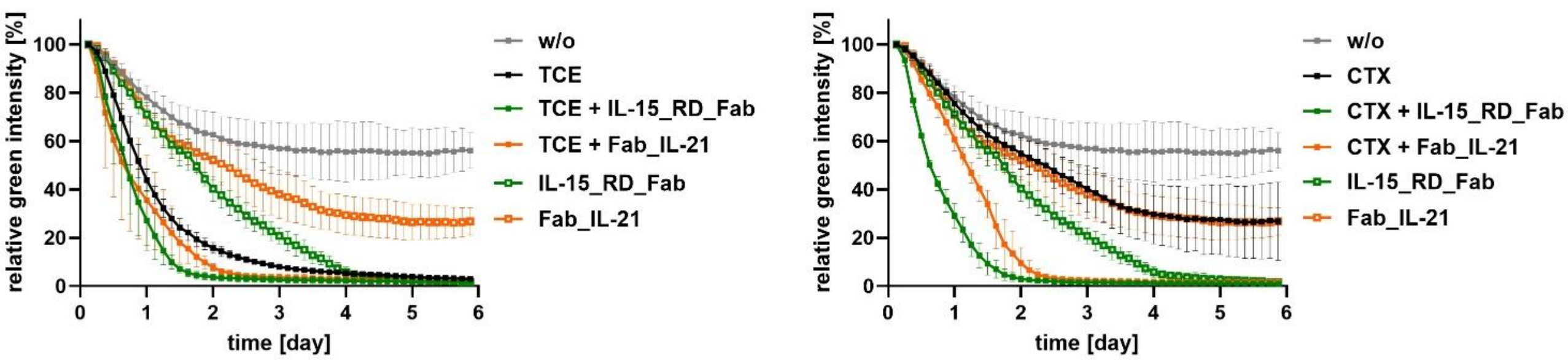

Supplementary Figure 7: Bifunctional fusion proteins enhance TCE- and Cetuximab-induced target cell killing. IKP-CAF-GFP spheroids were cocultured with resting PBMCs in presence of 0.4 nM fusion protein alone, and in combination with either TCE or Cetuximab at 0.25 nM. Cytotoxicity was monitored for 6 days via live-cell imaging (Incucyte), measuring green fluorescence reduction. Data of spheroids were normalized to the 3 h value. Graphics show mean ± SD, n=3, independent experiments using PBMC from the same donor with blockshift correction.
