# Supplementary material for: Trifunctional antibody fusion proteins for targeted co-delivery of 4-1BBL with IL-15 or IL-21 to enhance antitumor immune responses
Source: Front Immunol. 2026 Oct 7;17:1903525. doi: 10.3389/fimmu.2026.1903525 (PMC13683726; doi:10.3389/fimmu.2026.1903525)
Supplement: Supplementary file 9 [file DataSheet8.pdf]

# *Supplementary Material*

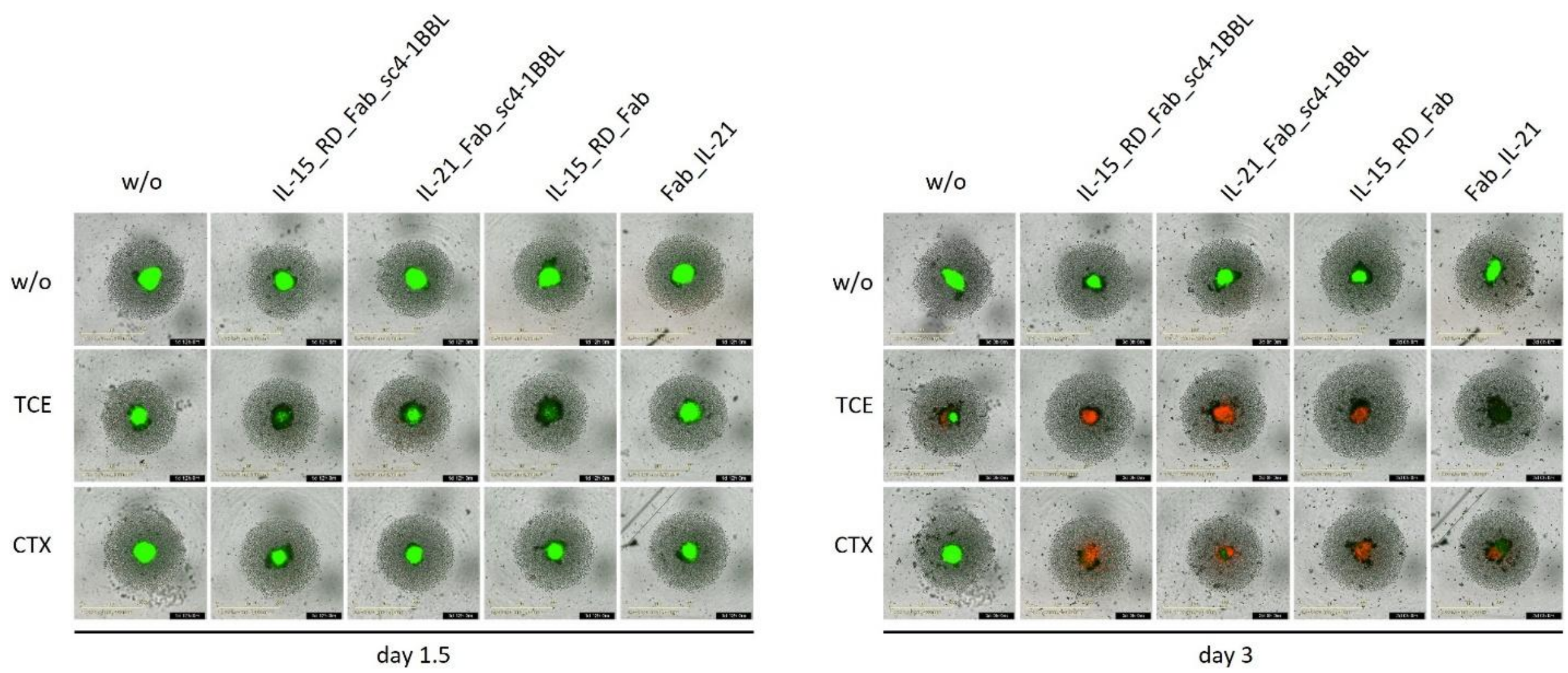


**Supplementary Figure 8:** Bi- and trifunctional fusion proteins enhance TCE- and Cetuximab-induced target cell killing. IKP-CAF-001 (GFP+) spheroids were cocultured with resting PBMCs in presence of 0.4 nM fusion protein alone, and in combination with either TCE or Cetuximab at 0.25 nM. Cytotoxicity was monitored for 6 days via live-cell imaging (Incucyte), measuring GFP fluorescence reduction (green) and 7-AAD (red). Representative images of day 1.5 and day 3 from 3 independent experiments using PBMC from the same donor, with 1-2 spheroids per condition per experiment.
