# Supplementary material for: Trifunctional antibody fusion proteins for targeted co-delivery of 4-1BBL with IL-15 or IL-21 to enhance antitumor immune responses
Source: Front Immunol. 2026 Oct 7;17:1903525. doi: 10.3389/fimmu.2026.1903525 (PMC13683726; doi:10.3389/fimmu.2026.1903525)
Supplement: Supplementary file 10 [file DataSheet9.pdf]

Supplementary Material

Table 1 Production and binding properties of bi- and trifunctional antibody fusion proteins

**Protein**	**MW [kDa]**	**Yield [g/L]**	**EC_{50} ± SD [nM]**<br>**Binding to B16-FAP cells**
IL-15_RD_Fab_sc4-1BBL	133.6 (48.1 + 85.5)	1.6	8.1 ± 2.4
IL-21_Fab_sc4-1BBL	126.3 (40.8 + 85.5)	4.1	8.7 ± 1.6
Fab_sc4-1BBL	110.2 (24.7 + 85.5)	3.1	3.0 ± 0.6
IL-15_RD_Fab	72.2 (48.1 + 24.1)	3.2	5.1 ± 3.0
IL-21_Fab	64.9 (40.8 + 24.1)	4.5	4.2 ± 0.6
